# Rational engineering unlocks the therapeutic potential of WHP1: A revolutionary peptide poised to advance wound healing

**DOI:** 10.1371/journal.pone.0323363

**Published:** 2025-05-14

**Authors:** Patcharin Khajornpipat, Onrapak Reamtong, Ratchaneewan Aunpad

**Affiliations:** 1 Graduate Program in Biomedical Sciences, Faculty of Allied Health Sciences, Thammasat University, Pathum Thani, Thailand; 2 Department of Molecular Tropical Medicine and Genetics, Faculty of Tropical Medicine, Mahidol University, Bangkok, Thailand; University of Waterloo, CANADA

## Abstract

Treatment of chronic or non-healing wounds has faced a considerable clinical challenge and impose several detrimental effects on individuals, society, the healthcare system, and the economy. Bioactive peptides have been employed to accelerate wound healing in active wound treatment efficiently and effectively. In the current study, a novel wound-healing peptide, WHP1, was designed from 23 existing wound-healing peptides by a rational template-assisted approach. It demonstrated the ability to enhance migration and proliferation of human keratinocyte cell lines (HaCaT) without exhibiting cytotoxic effects on human red blood cells and HaCaT cells. By quantitative proteomic analysis, WHP1 exerted a multifaceted role on diverse cellular processes in human keratinocyte. Notably, it increased the expression of intracellular proteins of HaCaT cells involved in cell cycle regulation and focal adhesion, including centromeric histone H3 variant CENPA, ubiquitin-conjugating enzyme E2 C, thyroid receptor-interacting protein 6, and ribosomal components essential for cell adhesion and migration. WHP1 upregulated the key enzyme glyceraldehyde-3-phosphate dehydrogenase, orchestrating metabolic biosynthesis particularly glycolysis, cell cycle regulation, and cytoskeletal processes. An intriguing observation was the antioxidant activity of WHP1, protecting cells from reactive oxygen species-induced senescence. This is consistent with the upregulation of GAPDH expression and reduction of histone H2A.J levels. WHP1 also stimulated macrophages to secrete transforming growth factor-β (TGF-β), a crucial growth factor necessary for the remodeling phase of wound healing. This investigation highlighted the feasibility of rational design to create novel wound-healing peptides. Such advancements hold promise for improving patients’ quality of life and elevating the standard of care in contemporary healthcare.

## Introduction

The skin is the largest human organ by surface area, covering approximately 2 m^2^ and weighing up to 10 kg in adults. One crucial function is to serve as a defensive barrier against foreign pathogens and environmental threats [1,2]. Upon injury, different types of cells in the skin layers must synchronize to initiate the wound healing process. This involves overlapping stages, encompassing hemostasis, inflammation, proliferation and remodeling [3,4]. Hemostasis is the body’s initial response to injury, encompassing vasoconstriction, platelet attachment, platelet aggregation, and blood clot formation [3–6]. Concurrently, activated macrophages at the injury site start the inflammation stage by releasing reactive oxygen species (ROS), cytokines, and growth factors that are crucial for combating infection, removing cellular debris, and facilitating cellular migration. As the healing process progresses into the proliferation stage, macrophages transition to an alternatively activated macrophages, secreting anti-inflammatory cytokines and growth factors that promote granulation tissue formation, fibroblast and keratinocyte proliferation, and angiogenesis [3,4,7–9]. This stage is marked by intense cellular proliferation and migration, working to cover and fill the wound [5,7]. Subsequently, collagen rearrangement into thicker bundles perpendicular to wound margins occurs, termed remodeling, which enhances wound contraction and tensile strength [7]. Failure in this complex repair process can result in delayed healing and chronic wound development [10,11].

Chronic wounds are defined by skin barrier defects and the inability to regain anatomical and functional integrity in a timely manner. These wounds continue to be a challenging problem in the healthcare system, causing concern among patients and clinicians globally [12]. It has been estimated that globally 20 million people suffer from chronic wounds [11,13]. The multiple factors which can hinder the healing process include aging, obesity, infection, and comorbidities. They result in heightened and persistent pro-inflammatory cytokines, senescent cells, and diminished migratory capacity and proliferation rates [11,14]. Under Medicare, a US health insurance program, wound treatment expenses were estimated in 2018 to be between 28 and 97 billion USD [15]. Given the substantial costs and patient distress, advancements in treatment strategies are imperative. Regrettably, existing treatments often fail to fully restore skin function and may even end in amputation. Hence, there is a pressing need for effective interventions to expedite the healing of acute injuries and manage non-healing wounds [10,11,16].

A promising strategy for managing chronic wounds is topical care. This is categorized as traditional, advanced or active wound care, based on its therapeutic purpose. The active wound care used for poorly healing wounds employs treatments such as proteins and peptides to stimulate wound recovery [17]. Epidermal growth factor (EGF) has been widely utilized in wound management and regenerative medicine for several decades. In the management of diabetic foot ulcers, the combination of recombinant human EGF (rhEGF) along with conventional therapies stands as a promising strategy to promote both partial and complete healing, and thereby prevent the necessity for extremity amputation [18,19]. This example underscores the therapeutic efficacy of peptides in enhancing wound healing. Nonetheless, the application of this compound is limited due to its susceptibility to rapid deactivation and relatively high cost [18,19].

Short bioactive peptides (BPs) represent alternative therapeutic agents in the treatment of chronic wounds. A number have been shown to have potent and diverse therapeutic properties, low toxicity, and diminished allergenicity compared to currently used therapeutic proteins. They also exhibit longer shelf life and strong stability, due to their minimal inclusion of higher-order structure, which hinders denaturation and makes them more cost-effective. Moreover, peptides have a low tendency to accumulate in the body, allowing them to be used in various manufacturing industries [20–22]. They are small organic compounds (fewer than 50 amino acid residues) and are sourced from a wide range of origins [20,21,23]. BPs facilitate cellular migration and proliferation through the activation of intracellular signaling pathways. They promote re-epithelialization, granulation tissue formation, and the deposition of extracellular matrix (ECM), ultimately helping to restore wound integrity. Furthermore, these peptides stimulate immune cells, particularly macrophages, to secrete cytokines and growth factors, thus making the micro-environment more conducive to accelerated wound healing [24,25]. Carretero et al. explored the wound healing ability of peptide LL-37 [26]. It promotes keratinocyte migration by activating Snail and Slug transcription factors, stimulating matrix metalloproteinases, and induces the mitogen-activated protein kinase (MAPK) and the phosphatidylinositol 3-kinase (PI3K)/Akt signaling via formyl peptide receptor-like 1 (FPRL1) activation. *In vivo*, LL-37 enhances granulation tissue formation and re-epithelialization in wounds of diabetic mice. It also demonstrates immunomodulatory effects by enhancing chemotaxis of CD4 T lymphocytes [17,24,26,27]. Ot-WHP, previously discovered to have immunomodulatory properties (such as increasing TGF-β levels), also promotes collagen deposition and accelerates the healing process *in vivo* [10].

A fundamental step in the pursuit of effective peptides involves their rational design, particularly through use of template-assisted approach [20,21,28]. Residues and motifs vital for activity and selectivity are identified by sequence alignment. Prior studies have successfully utilized this method to design potent BPs [10,29,30]. For instance, the peptide PA-13 was rationally designed from α-helical cathelicidin and aurein through a template-modified technique. It demonstrates enhanced antimicrobial activity along with reduced toxicity [30]. We used this approach to design a novel wound-healing peptide, WHP1, and here describe that process and the assessment of the peptide. This study further advances the rational design of synthetic peptide for wound healing and gains insights into its mechanism of action, providing a blueprint to fully unlock the therapeutic potential.

## Materials and methods

### Peptide design and *in silico* sequence analysis

Drawing inspiration from template-modified antimicrobial peptides [30], 23 wound-healing peptide sequences sourced from the Antimicrobial Peptide Database (APD; http://aps.unmc.edu/AP/main.php) were aligned using ClustalX2 [31]. The most conserved sequences were identified and used as a template for the design of a novel wound-healing peptide, WHP1. The physicochemical properties and two-dimensional (2D) chemical structure of this peptide was analyzed using the online bioinformatics programs, at PepDraw server (available at https://pepdraw.com/) [32]. Its 3D chemical structure was predicted using PEP-FOLD3 which was available at http://bioserv.rpbs.univ-paris-diderot.fr/services/PEP-FOLD3 [33]. The isoelectric point (pI) of the peptide was evaluated using the Expasy pI/Mw tool, accessible at https://web.expasy.org/compute_pi/ [34]. Additionally, the three-dimensional (3D) configuration of this novel peptide was predicted using the Iterative Threading ASSEmbly Refinement (I-TASSER) tool (accessible at http://zhanglab.ccmb.med.umich.edu/I-TASSER/) [35].

### Peptide synthesis

WHP1 peptide was synthesized by ChinaPeptides (China) using a standard 9-fluorenylmethoxycarbonyl (Fmoc) solid-phase method. Subsequently, purification was conducted on a C18 column of trifluoroacetate (TFA) salts, utilizing reverse-phase high-performance liquid chromatography (RP-HPLC). Analytical RP-HPLC confirmed that the peptide purity surpassed 98%. The peptide sequence was verified using electrospray ionization mass spectrometry (ESI-MS). The peptide was reconstituted in deionized water (DI; 10 mg/ml stock concentration) and stored at -20°C.

### Determination of hemolytic activity of peptides

The hemolytic activity of WHP1 was investigated by quantifying hemoglobin release from human red blood cells (hRBCs) following peptide exposure [36]. Fresh hRBCs of blood type O, collected from healthy adult volunteers into polycarbonate tubes containing EDTA anticoagulant. Following centrifugation at 1,000 × g for 5 min, the erythrocytes were washed with sterile phosphate-buffered saline (PBS; Caisson Laboratories, Utah, USA) until the supernatant turned clear. The cells were then resuspended in PBS to achieve a 2% (v/v) erythrocyte suspension, as previously described [37]. To determine the hemolytic activity of WHP1, hRBC suspension was incubated at 37°C for 1 h with equal volumes peptide, at concentrations ranging from 0.98 to 250 µg/ml. Subsequently, the hRBCs-peptide mixtures were centrifuged at 1,000 × g for 10 min prior to transfer of supernatant to 96-well plates. Hemoglobin release was quantified in triplicate by recording absorbance values at 405 nm. Untreated red blood cells served as negative controls, while Triton X-100 was used as a positive control. LL-37 was included for comparison of its hemolytic activity to that of WHP1. The hemolysis percentage was calculated using the equation (eq. 1):


$$\text{Hemolysis }\left(\text{\textbackslash{}nonumber\textbackslash{} \textbackslash{}\textbackslash{}\%}\right)\text{ = }\left[\frac{\left({\text{A}}_{\text{Sample}}\text{-}{\text{A}}_{\text{Blank}}\right)}{\left({\text{A}}_{\text{TritonX-100}}\text{-}{\text{A}}_{\text{Blank}}\right)}\right]\text{×100}$$ (1)

In eq. 1, A_Blank_ represents the absorbance of hRBCs with PBS, acting as the negative control (0% hemolysis). A_TritonX−100_ signifies the absorbance of 0.1% Triton X-100, utilized as the positive control (100% hemolysis).

### Ethics statement

In accordance with ethical standards for studies with human subjects, the experimental protocol was approved by the Ethics Committee of Thammasat University (COA No. 022/2567). Volunteer recruitment was conducted from 01/04/2024–31/08/2024. All study subjects gave written informed consent.

### Cell culture and growth conditions

Human HaCaT keratinocyte cell lines (RRID:CVCL_0038) were cultivated in Dulbecco’s modified Eagle’s medium (DMEM) (Gibco, USA), supplemented with 10% heat-inactivated fetal bovine serum (FBS) (Gibco, USA) and 100 U-100 μg/ml penicillin-streptomycin (Gibco, USA). Meanwhile, the human leukemia monocytic cell line (THP-1) (ATCC TIB-202) was cultured in Roswell Park Memorial Institute (RPMI) medium (Gibco, USA), supplemented with 2 mM glutamine (Gibco, USA) and 10% heat-inactivated FBS. Cell cultures were maintained at 37°C within a humidified incubator under an atmosphere of 5%CO_2_.

### Determination of cytotoxicity of peptides

Cytotoxicity of WHP1 exposure to normal human HaCaT keratinocyte cell lines was conducted using the thiazolyl blue tetrazolium bromide (MTT) colorimetric assay, as previously described [38]. After culturing the cells at 37°C in DMEM under 5% CO_2_ and high humidity, they were seeded onto sterile 96-well plates at a density of 2.5 × 10^4^ cells/well. They were then treated with varying concentrations of WHP1, ranging from 0.98 to 250 µg/ml, for a duration of 24 h. Following treatment, they were incubated for 3 h at 37°C in 100 µl of 0.4 mg/ml MTT (Invitrogen, UK). After removing the supernatant, dimethyl sulfoxide (DMSO) (Thermo Fisher Scientific, UK) was added. The absorbance was subsequently quantified at 570 nm employing a microplate reader, with untreated cells serving as negative controls. LL-37 was used for comparison of its cytotoxicity to that of WHP1. The percentage of cytotoxicity was calculated using the following equation (eq. 2):


$$\text{Cytotoxicity }\left(\text{\textbackslash{}nonumber\textbackslash{} \textbackslash{}\textbackslash{}\%}\right)\text{ = }\left(\frac{{\text{A}}_{\text{Treated}}}{{\text{A}}_{\text{Control}}}\right)\text{×100}$$ (2)

In eq. 2, A_Treated_ denotes the absorbance observed in peptide-treated cells, while A_Control_ indicates the absorbance in cells without peptide treatment.

### Scratch wound closure assay

A scratch wound closure assay was used to assess the capacity of WHP1 to induce cell migration [39]. Initially, HaCaT cells were seeded onto 24-well plates at a density of 3 × 10^5^ cells/well and allowed to form confluent cell monolayers. Following overnight incubation, cells were treated for 1 h with serum-free DMEM containing 10 µg/ml of mitomycin C (MMC) (Sigma-Aldrich, USA) to arrest cell proliferation. Afterwards, artificial wounds were created by vertically scratching the cell monolayer using a sterile micropipette tip. The scratched cells were rinsed twice with PBS to remove any detached cells prior to treatment with WHP1 peptide. PBS was used as a negative control, while EGF acted as a positive control in the assay. LL-37, a peptide well-known for its wound healing and immunomodulatory activities [17,24,26,27], was also included for comparison to WHP1. Images of the wound area in the scratched cell monolayers at 0 and 24 h were captured using a Lionheart FX automated microscope (Agilent Technologies, USA). The percentage area recovered, indicative of cell migration activity, was calculated using Image J software.

### Cell proliferation assay

A colorimetric assay was employed to investigate cell proliferation, aiming to determine whether wound closure was facilitated by enhanced cell proliferation following exposure to the WHP1 [40]. HaCaT cells were seeded into 96-well plates at a density of 5 × 10^3^ cells/well. Following an overnight incubation, the cells were exposed to DMEM, PBS, EGF, LL-37, and varying concentrations of WHP1 at 24, 48, and 72-h intervals. Then, 100 µl of 0.4 mg/ml MTT solution was added. After a 3-h incubation at 37°C, 100 µl of DMSO was introduced, and the absorbance was assessed at a wavelength of 570 nm. PBS was employed as the negative control, EGF as the positive control, and LL-37 was included for comparison with WHP1.

### Determination of free radical scavenging activity

#### Determination of DPPH radical scavenging activity.

The 2,2-diphenyl-1-picrylhydrazyl (DPPH^•^) radical scavenging activity of WHP1 was assessed using a previously established procedure [41]. One hundred μl of freshly prepared DPPH reagent (Sigma-Aldrich, USA) was incubated with WHP1 at concentrations ranging from 0.98 to 250 μg/ml for 30 min in the dark. Subsequently, absorbances were measured at 517 nm using a microplate reader. Using the equation below (eq. 3), the percentage decolorization representing DPPH scavenging was calculated. The IC_50_ values indicating the concentration at which WHP1 effectively reduces 50% of free radical activity were determined using GraphPad Prism 7.00 software. L-glutathione served as the positive control and deionized water was as the negative. LL-37 was also included for comparison with WHP1.


$$\text{Decolorization }\left(\text{\textbackslash{}nonumber\textbackslash{} \textbackslash{}\textbackslash{}\%}\right)\text{ = }\left[1-\left(\frac{{\text{A}}_{\text{Treated}}}{{\text{A}}_{\text{Control}}}\right)\right]\text{×100}$$ (3)

In eq. 3, A_Treated_ denotes the absorbance observed in peptide incubated with the reagent, while A_Control_ indicates the absorbance of reagent without peptide.

#### Determination of ABTS radical scavenging activity.

The 2,2′-azino-bis (3-ethylbenzthiazoline-6-sulphonic acid) (ABTS^•+^) radical scavenging activity of WHP1 was assessed using a previously described protocol [42]. To initiate the experiment, an ABTS (Sigma-Aldrich, USA) stock solution was prepared by mixing it with potassium persulfate (Sigma-Aldrich, USA), generating ABTS^+^ radical cations. This mixture was then allowed to incubate at 4°C for 12–16 h in the dark. Afterwards, the mixture was diluted with PBS buffer until it reached an absorbance of 0.7 at 734 nm. Following this preparation step, 50 μl of WHP1 (with concentrations varying from 0.98 to 250 μg/ml) was added to 200 μl of the ABTS mixture, and absorbance was promptly measured at 734 nm utilizing a microplate reader. The decolorization percentage was calculated using the below equation (eq. 4), and IC_50_ values were determined using GraphPad Prism 7.00 software.


$$\text{Decolorization }\left(\text{\textbackslash{}nonumber\textbackslash{} \textbackslash{}\textbackslash{}\%}\right)\text{ = }\left(\frac{{{\text{A}}_{\text{Control}}\text{-A}}_{\text{Treated}}}{{\text{A}}_{\text{Control}}}\right)\text{×100}$$ (4)

In eq. 4, A_Treated_ denotes the absorbance observed in peptide incubated with the reagent, while A_Control_ indicates the absorbance with reagent without peptide.

### Growth factor and cytokine measurements

The impact of WHP1 on cytokine and growth factor secretion *in vitro* was assessed using ELISA. The THP-1 cell line was seeded at a density of 5 × 10^5^ cells/well in 24-well culture plates. Monocyte differentiation into macrophages was induced by a 24-h incubation period with 50 ng/ml of phorbol 12-myristate 13-acetate (PMA) (Sigma-Aldrich, USA). The cells were then treated with either WHP1 or PBS for a duration of 24 h. Subsequently, the culture supernatants were collected and centrifuged at 10,000 × g for 10 min at 4°C. The quantification of TNF-α, TGF-β1, and IL-10 levels was performed using commercial ELISA kits (DuoSet ELISA Kit, R&D Systems, USA), following the manufacturer’s instructions. LL-37 was used to assess its immunomodulatory activity compared to WHP1. Each experiment was independently performed in triplicate [10,43]. The equation below (eq. 5) was used to calculate the fold change of cytokine and growth factor levels [44].


$$\text{Fold change relative to negative control = }\left(\frac{{{\text{Mean}}_{\text{Treated}}\text{-Mean}}_{\text{Control}}}{{\text{Mean}}_{\text{Control}}}\right)$$ (5)

In eq. 5, Mean_Treated_ denotes the mean value of specimen from peptide-treated cells, while Mean_Control_ indicates the mean value of specimen from cells without peptide treatment.

### Proteomic analysis

Proteomic analyses were employed to investigate protein changes in HaCaT cells following WHP1 peptide treatment at a concentration of 62.5 μg/ml. After a 24-h treatment with WHP1, the HaCaT cell lines were incubated at 50°C for 10 min and subsequently sonicated at 20 kHz for 5 sec while kept on ice. After undergoing centrifugation at 15,000 × g, supernatants were collected and precipitated using a mixture of ice-cold trichloroacetic acid/acetone/methanol. The precipitated cells were then redissolved in a buffer containing 0.5% RapiGestSF, 10 mM NaCl, and 10 mM ammonium bicarbonate. To ensure complete solubilization, the cells were lysed by sonication on ice at 20 kHz for 2 sec. Subsequently, 50 µg of total protein from the lysed cells were processed for gel-free digestion. In brief, the reduction of sulfhydryl bonds was carried out using 2 mM tris(2-carboxyethyl)phosphine (TCEP) in 5 mM ammonium bicarbonate at 45°C for 2 h. This was followed by sulfhydryl alkylation with 10 mM IAA in 5 mM ammonium bicarbonate at room temperature for 45 min in the dark. Finally, the cells underwent enzymatic digestion with sequencing-grade Trypsin/LysC for 16 h. The resulting tryptic peptides were reconstituted in a solution of 0.1% formic acid before being analyzed using liquid chromatography with tandem mass spectrometry (LC-MS/MS) [45].

Proteomics analysis was conducted using a SciEx high-resolution 6600 + TripleTOF^TM^ (AB-Sciex, Canada) coupled with an UltiMate 3000 nanoLC System (Thermo Fisher Scientific, USA). Reconstituted tryptic peptides were injected with a sample volume of 5 µl at a concentration of 100 µg/µl. The LC conditions were set up as follows: Mobile phases A and M consisted of 0.1% formic acid in water and 90% acetonitrile with 0.1% formic acid, respectively. The samples were directly loaded onto an analytical C18 column (reverse-phase chromatography) with a particle size of 2 µm and dimension of 75 µm x 25 cm. Separation was conducted over a duration of 135 min with a constant flow rate of 300 nl/min using a 135-min gradient for a single injection. The column temperature was maintained at 55°C throughout the process. During the data-dependent acquisition (DDA) mode of mass spectrometry, MS scans were performed over a mass range of 400–1600 m/z. Peptide ions, specifically the top 20 with charge states between 2 and 5 in positive mode, were selected for fragmentation, employing a dynamic exclusion time of 12 sec. The raw MS spectra (.wiff files) were processed and annotated with corresponding protein sequences through the utilization of the Paragon™ Algorithm in ProteinPilot™ Software. The reference proteome of Homo sapiens, obtained from the reviewed UniprotKB database and compiled in FASTA format, was downloaded on February 10, 2024. Proteins were identified using stringent criteria to ensure accuracy and reliability: only proteins meeting the criteria of a false discovery rate (FDR) ≤ 1% and identified by at least 10 peptides were included in the final protein lists [45].

The FDR for peptide and protein identification was set at 1%. Label-free quantification identified significant differences, defined by a fold change of 2 or more, and a *p*-value below 0.05, determined using t-test. Proteins exhibiting these alterations underwent protein-protein interaction analysis using STRING software (http://string-db.org/) [46]. Statistical analyses were conducted using MetaboAnalyst 6.0 [47].

### Statistical analysis

The results were presented as the mean ± standard deviation (SD), with each experiment performed independently and repeated a minimum of three times. The comparison of differences between the control and experimental groups was conducted employing Student’s t-test. Statistical analyses were computed using GraphPad Prism version 7.0, encompassing one-way analysis of variance (one-way ANOVA) and Tukey’s post hoc test. Statistical significance was set at a *p*-value of less than 0.05 with a confidence level of 95%.

In the context of the proteomics, protein-level analyses were conducted using pairwise comparisons and one-way ANOVA. This analysis included two methods for correcting multiple tests: the Bonferroni correction and the Benjamini-Hochberg FDR correction, both executed using ProteinPilot™ software [47].

## Results

### Design and characteristics of novel wound healing peptide, WHP1

**Fig 1:**
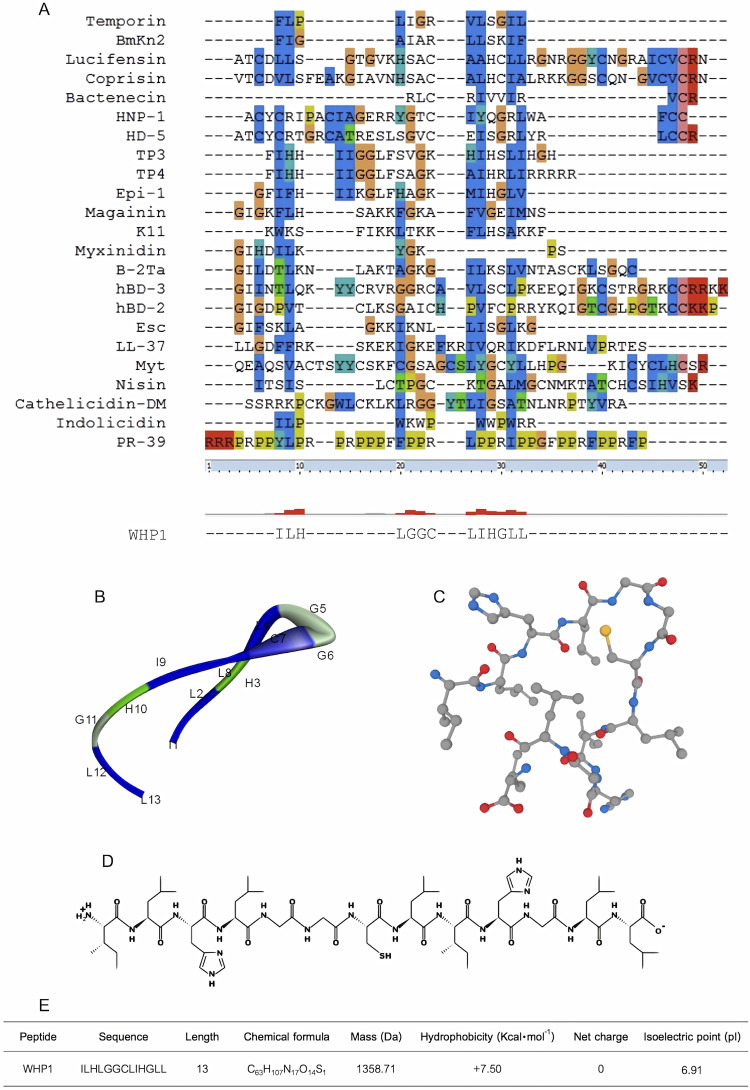
Design and evaluation of WHP1 peptide using *in silico* analysis. (A) Multiple sequence alignments of 23 peptides. The hydrophobic residues are depicted in blue, polar residues in green, negatively charged residues in magenta, and positively charged residues in red. Distinctive residues such as glycine, aromatic residues, and proline are highlighted in orange, cyan, and yellow, respectively. (B) A 3D ribbon diagram of WHP1. The residues with positive charge are represented in green, the hydrophobic residues in blue, and the glycine residues in light green. The numerical values are provided to indicate the positions of amino acid residues. (C) A 3D chemical structure model of WHP1. Elements of carbon, oxygen, nitrogen, and sulfur are represented by gray, red, blue, and yellow, respectively. (D) The 2D chemical structure of the WHP1 peptide. (E) The physicochemical characteristics of WHP1.

Employing a template-modified strategy, we have engineered a novel peptide, WHP1, characterized by the sequence ILHLGGCLIHGLL (Fig 1). This peptide was designed for wound healing applications through the alignment of sequences from 23 established peptides with known wound healing properties. The conserved sequence from this alignment influenced the rational design of WHP1, aiming to ensure its efficacy and specificity in facilitating wound repair. Through *in silico* analysis, the WHP1 peptide was predicted to adopt a random coil conformation with a hydrophobicity of + 7.5 Kcal⋅mol^-1^, a net charge of 0, and a pI of 6.91. The WHP1 peptide was chemically synthesized using these specified amino acid residues, and the mass of the synthesized peptide was subsequently confirmed via ESI-MS. The measured mass of the synthesized peptide closely matched the calculated value of 1358.7 Da, affirming its successful synthesis.

### The WHP1 peptide demonstrates potent wound-healing activity

**Fig 2:**
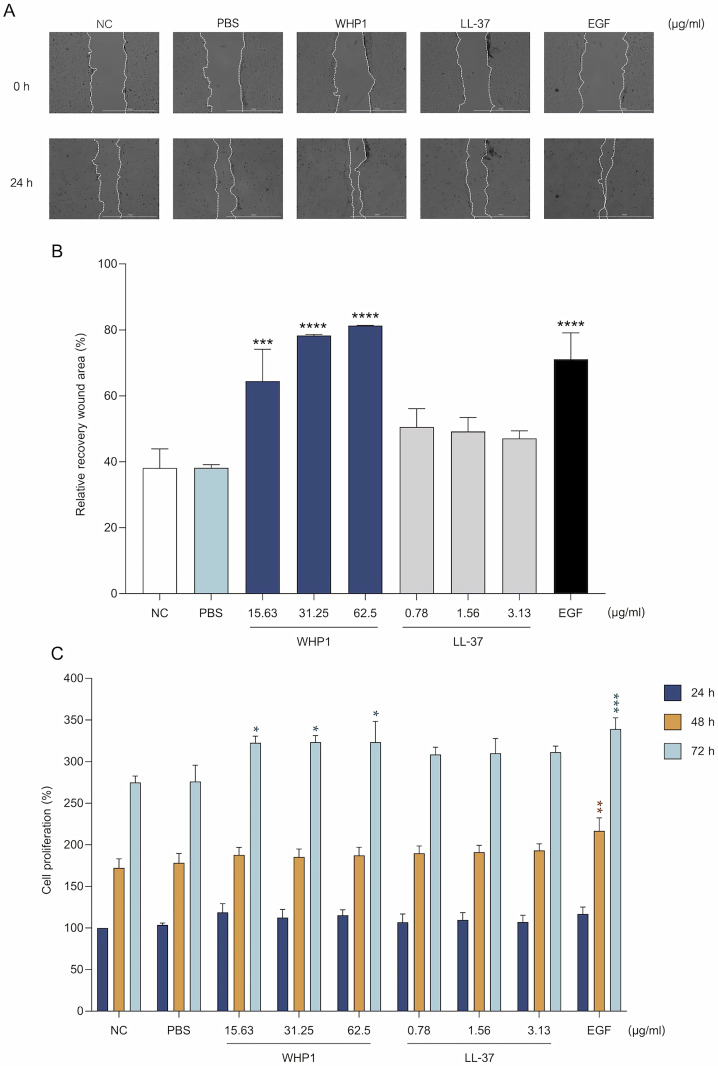
HaCaT cell migration and proliferation induced by WHP1. **(A)** The scratch wound area of HaCaT cells treated with WHP1, along with negative control (NC or culture media without peptide), PBS, LL-37, and EGF (25 ng/ml; positive control). **(B)** Percentage relative recovery of the wound area after treatment with WHP1 at different concentrations. **(C)** HaCaT proliferation after WHP1 treatment at various time points (24, 48 and 72 h), compared to the NC, PBS, LL-37, and EGF (25 ng/ml). *p < 0.05, **p < 0.01, ***p < 0.001, and ****p < 0.0001.

Keratinocyte proliferation and migration are integral to re-epithelialization and the acceleration of wound repair, and represent critical mechanisms in wound closure and the overall healing [48]. To elucidate the impact of WHP1 on HaCaT cell migration and wound closure, an *in vitro* scratch wound assay was conducted. Exposure to WHP1 at concentrations ranging from 15.63 to 62.5 μg/ml significantly enhanced cell migration into the wound area, with the most pronounced effect observed at 62.5 μg/ml, compared to negative controls. This response exhibited a dose-dependent relationship (Fig 2A and 2B). Remarkedly, the WHP1 demonstrated significantly greater efficacy in enhancing cell migration compared to EGF, a polypeptide known to promote wound healing [18,19]. Conversely, the wound closure activity of LL-37, a peptide known for promoting keratinocyte migration and wound healing both *in vitro* and *in vivo* [26], was not significantly greater than the control and PBS-treated groups.

WHP1 obviously enhanced HaCaT proliferation within the concentration range of 15.63 to 62.5 μg/ml. At a concentration of 62.5 μg/ml, proliferation at 72 h was more than 40% greater than controls. The treatment with LL-37 for 72 h also exhibited a substantial increase in HaCaT cell proliferation (over 30% at a concentration of 3.13 μg/ml) (Fig 2C). These findings underscore the potential of WHP1 to promote keratinocyte migration and proliferation, highlighting its potential use in wound healing applications.

### The WHP1 peptide exhibits no toxic effects

**Fig 3:**
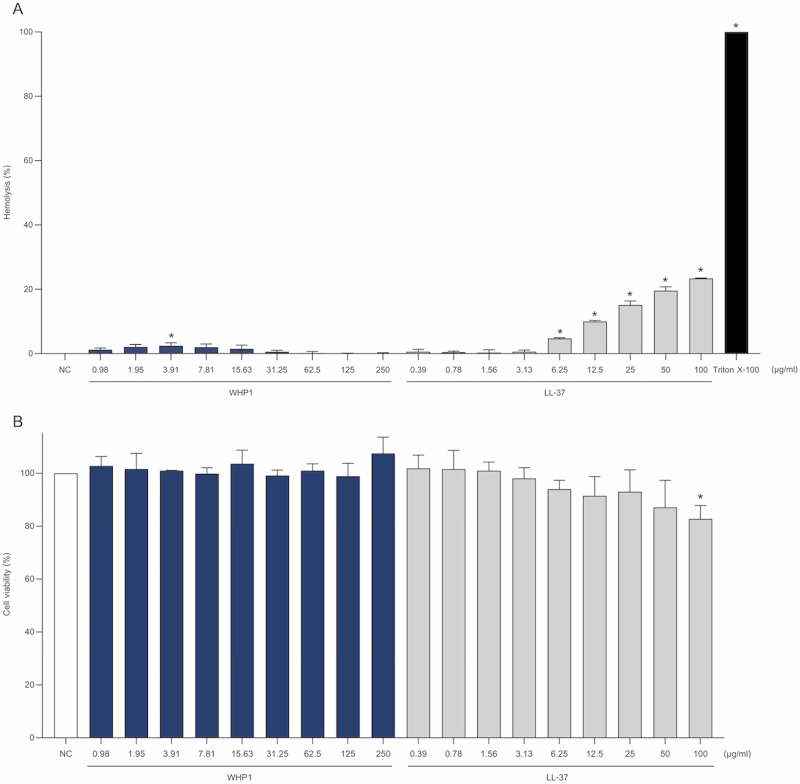
The toxic effects of WHP1. **(A)** The hemolytic activity of WHP1 peptide on human red blood cells, along with negative control (NC) or culture media without peptide, LL-37, and 0.1% Triton X-100. **(B)** The viability of HaCaT cells after treatment with WHP1 and LL-37 peptides. NC represents HaCaT cells without peptide treatment. *p < 0.05.

The hemolytic activity assay revealed that even at the highest concentration of 250 μg/ml, WHP1 caused no significant hemolysis of erythrocytes (Fig 3A). LL-37 displayed toxic effects at concentrations as low as 6.25 μg/ml, with toxicity exceeding 20% at 100 μg/ml (Fig 3A). The cytotoxicity of the WHP1 was assessed using viability of HaCaT cells, and it was found to be non-toxic across the concentration range of 0.98 to 250 μg/ml (Fig 3B). LL-37 demonstrated a notable reduction in HaCaT cell viability, which was dose-dependent and became statistically significant at 100 μg/ml. Additionally, the antimicrobial activity of WHP1 was assessed; however, no detectable antimicrobial effect was observed (Table S1 in S1 File).

### The WHP1 peptide displays substantial free radical scavenging activity

**Fig 4:**
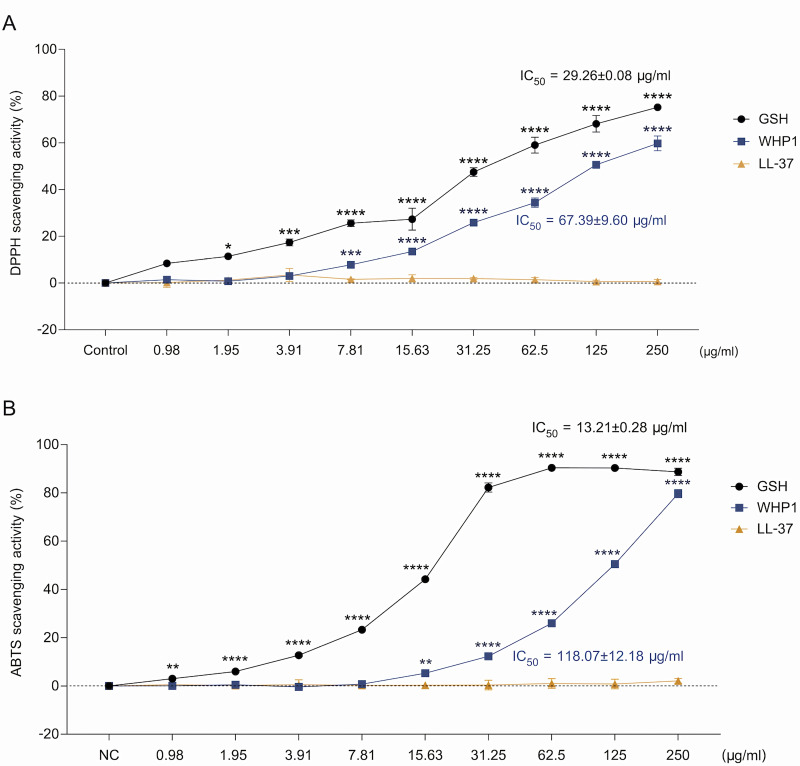
Radical scavenging activity of WHP1. **(A)** The DPPH scavenging activity of WHP1, with GSH as positive control and LL-37 as comparators. **(B)** The ABTS scavenging activity with GSH and LL-37. IC_50_ values are presented as the mean ± SD. *p < 0.05, **p < 0.01, ***p < 0.001, and ****p < 0.0001.

Antioxidant activities, particularly radical scavenging, are vital in counteracting the detrimental effects of free radicals on cellular integrity within wound sites, as these radicals can exacerbate cellular damage and impede the wound healing [7,49]. An antioxidant substance known as L-glutathione (GSH) was employed as a positive control in this experiment [50]. GSH exhibited potent antioxidant activity, with DPPH and ABTS radical scavenging IC_50_ values of 29.26 ± 0.08 µg/ml and 13.21 ± 0.28 µg/ml, respectively. Similarly, the WHP1 demonstrated strong antioxidant activity, with concentration-dependent inhibition of both DPPH and ABTS radical scavenging activities, showing IC_50_ values of 67.39 ± 9.60 and 118.07 ± 12.18 μg/ml, respectively (Fig 4). While the antioxidant activity of WHP1 is lower than that of the positive control, GSH, these results still suggest that WHP1 exhibits significant antioxidant potential, comparable to other antioxidant peptides [51,52], highlighting its promising therapeutic value.

### The WHP1 increases TGF-β secretion in THP-1-induced macrophages

**Fig 5:**
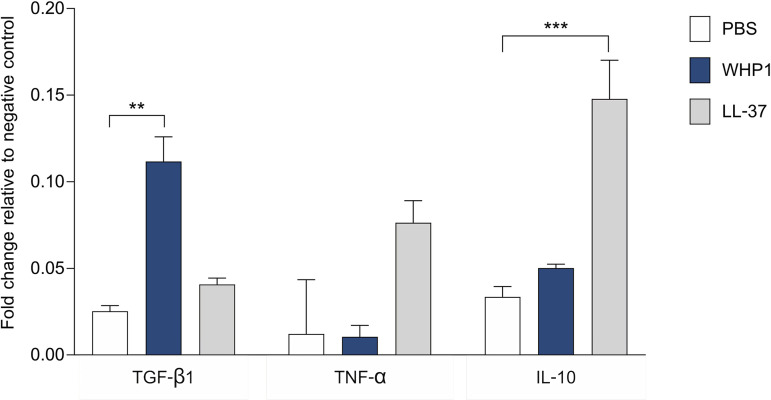
The secretion of cytokines after stimulation by WHP1 and LL-37 peptides. The bar graph illustrates the fold change in levels of TGF-β1, TNF-α and IL-10 secreted by THP-1-induced macrophages. **p < 0.01 and ***p < 0.001.

To elucidate the potential of WHP1 to enhance the production of cytokines and growth factors in the macrophage-driven microenvironments, THP-1 cells were induced to differentiate into macrophages and treated with peptide. Peptide WHP1 (at 62.5 μg/ml) was found to significantly stimulate a more than 4-fold increase in the production of the growth factor TGF-β1 compared to the PBS group (Fig 5). In contrast, the levels of TNF-α and IL-10 cytokines were unchanged. LL-37 (at 25 μg/ml) significantly elevated the level of IL-10 cytokine (by 4-fold) (Fig 5). Thus, WHP1 was shown to possess the capacity to stimulate the *in vitro* secretion of TGF-β1 by macrophages.

### The WHP1 peptide drives cell cycle progression and focal adhesion while suppressing senescence-related signaling

**Fig 6:**
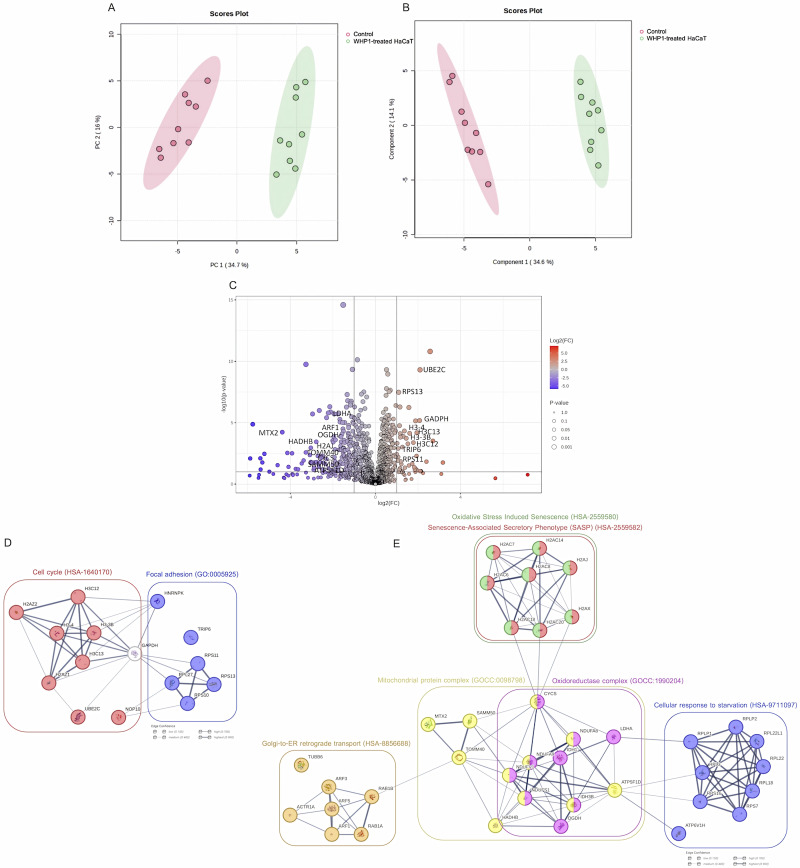
Proteomic analysis of protein expression in HaCaT cells following 24 h of WHP1 treatment. **(A)** Pairwise analysis of proteomic data from WHP1-treated HaCaT cells using principal component analysis. **(B)** Pairwise analysis of proteomic data from WHP1-treated HaCaT cells using partial least squares–discriminant analysis. **(C)** Volcano plots illustrating differential protein expression of HaCaT cells after treatment with WHP1. The horizontal lines denote *p*-values of 0.05, while the left and right vertical lines indicate fold changes of -2.0 and 2.0, respectively. **(D)** Protein-protein interaction analysis of upregulated proteins in WHP1-treated HaCaT cells using STRING v11.5. Significant gene ontologies were enriched; those for focal adhesion highlighted in purple, and for cell cycle in red. **(E)** Protein-protein interaction analysis of downregulated proteins in WHP1-treated HaCaT cells. Significant gene ontologies were enriched, highlighted in red, green, purple, orange, pink, and yellow.

To gain a comprehensive understanding of the mechanisms underlying how the WHP1 peptide promotes migration and proliferation of HaCaT cells, proteomic analysis using LC-MS/MS was conducted. Pairwise comparisons of proteomic data using principal component analysis (PCA) and partial least squares discriminant analysis (PLS-DA) revealed significant differences between untreated and WHP1-treated groups. PCA was conducted to reduce the dimensionality of the proteomic data and visualize variations. The first principal component (PC1) accounted for 34.7% of the total variation, while the second principal component (PC2) described 16.0%. The scores plot revealed distinct clustering for untreated and treated groups (Fig 6A). The clear separation between these groups suggested that WHP1 treatment induced substantial changes in the protein expression profiles of HaCaT cells. The PCA findings were corroborated by the PLS-DA results. Its scores plot (Fig 6B) demonstrates a clear separation between the untreated and treated groups, underscoring the distinct impact of WHP1 on the proteome of HaCaT cells. The first component (Component 1) accounted for 34.6% of the variance, effectively distinguishing between the untreated and WHP1-treated groups, while the second component (Component 2) accounted for an additional 14.1%.

A volcano plot visually illustrates the magnitude of fold change and statistical significance, offering insights into the impact of WHP1 treatment on proteomic expression relative to that of the untreated group (Fig 6C). Applying a fold-change threshold of ≥ 2 and a *p*-value cutoff of ≤ 0.05 allowed the identification of proteins with substantial expression changes. Proteins located above the horizontal threshold line were deemed significantly upregulated (n = 36), while those below the lower threshold line were considered significantly downregulated (n = 150).

A functional enrichment analysis of the identified differential proteins was conducted utilizing the protein-protein interaction network within STRING, as illustrated in Fig 6D and 6E. This analysis unveiled distinctive patterns among the upregulated and downregulated proteins. The network of upregulated proteins exhibited enrichments in interactions associated with crucial cellular processes such as the cell cycle (HSA-1640170) and focal adhesion (GO: 0005925). Within the cell cycle network, important regulators such as the centromeric histone H3 variant CENPA (1.45-fold) and ubiquitin-conjugating enzyme E2 (UBE2C) (1.09-fold) were prominently displayed (Table S2 in S1 File). The focal adhesion network comprised crucial proteins like thyroid receptor-interacting protein 6 (TRIP6) (1.02-fold) and various ribosomal components. Central to the interaction network, a significant upregulation (2.08-fold) of glyceraldehyde-3-phosphate dehydrogenase (GAPDH) expression was observed, highlighting its interactions with both the cell cycle and focal adhesion networks. These findings emphasized the functions of WHP1 in the promotion of cellular proliferation and migration.

Conversely, the downregulated proteins were associated with several key components and pathways. These included the senescence-associated secretory phenotype (SASP) (HSA-2559582) and oxidative stress-induced senescence (HSA-2559580), which notably included histone H2A variants, especially histone H2A.J (1.42-fold) (Table S3 in S1 File). Downregulated proteins were also linked to Golgi-to-ER retrograde transport (HSA-8856688), involving ADP-ribosylation factor (ARFs) (1.53-fold), Ras-related protein (Rab)-1A (4.14-fold) and -1B (1.61-fold), alpha-centractin (ARP1) (1.71-fold), and tubulin beta-6 chain (TUBB6) (1.67-fold). Additionally, pathways related to the oxidoreductase complex (GOCC: 1990204) and the mitochondrial protein complex (GOCC: 0098798) were downregulated. These include NADH dehydrogenase (complex I) (6.05-fold), metaxin-2 (MTX2) (4.38-fold), trifunctional enzyme subunit beta (TP-beta) (2.79-fold), NADH-ubiquinone oxidoreductase (complex I) (2.52-fold), cytochrome c (Cytc) (1.47-fold), 2-oxoglutarate dehydrogenase complex (OGDHC) (1.26-fold), isocitrate dehydrogenase (IDH) (1.23-fold), l-lactate dehydrogenase A chain (LDH-A) (1.14-fold), ATP synthase subunit delta (1.08-fold), sorting and assembly machinery component 50 homolog (SAMM50) (1.04-fold), and mitochondrial import receptor subunit TOM40 homolog (TOMM40) (1.00-fold). The pathway related of the cellular response to starvation (HSA-9711097) was also downregulated, involving the V-type proton ATPase subunit H (6.29-fold) and various ribosomal proteins. These observations imply a potential suppression of senescence-related signaling.

## DIscussion

Chronic wounds fail to follow the orderly stages of healing and impose significant humanistic and economic burdens on individual patients and society as a whole [13,53,54]. These wounds are also more frequent and more difficult to treat due to aging populations and the rising prevalence of diabetes and obesity. In chronic diabetic wounds, hyperglycemia activates NADPH oxidases, increasing ROS production and impaired fibroblast and keratinocyte function. This hinders re-epithelialization, which is crucial for wound coverage and infection prevention. Additionally, tissue recovery is delayed by increased pro-inflammatory molecules (like TNF-α) and decreased TGF-β [55,56]. Topical therapies for wound treatment are particularly attractive due to their localized targeting, minimal systemic accumulation, cost-effectiveness and ease of use. Peptides have significant potential as lead compounds for innovative therapeutic strategies and regenerative interventions in poorly healing wounds. According to recent projections, sales of synthetic peptides may have reached more than $400 million by 2023 [17,57].

Using a rational template-modified approach, a novel wound-healing peptide, WHP1, was successfully designed. The presence of cysteine and the specific motifs, such as the GxxxxG, in a peptide sequence is thought to be crucial for its biological activity, potentially facilitating accelerated wound healing [58–60]. The inclusion of cysteine in the peptide sequence of WHP1 provides it the ability to form intermolecular disulfide bonds, facilitating peptide dimerization through cysteine linkages [61]. Dimer self-assembly is a common biophysical phenomenon occurring in various cellular compartments. This process enhances protein stability and plays a crucial role in the growth and development of organisms. Dimerization also significantly regulates various cellular pathways, including signal transduction [62]. The pro-healing peptide OA-GP11d is a homodimer containing cysteine within its sequence [61,63]. It effectively promotes keratinocyte cell-scratch healing by activating the MAPK signaling pathway. The frog skin peptide CW49A, which also includes cysteine, promotes wound healing in full-thickness dermal murine models, both normal and diabetic. Another intramolecular disulfide bridge peptide, termed OM-LV20, not only enhances the migration and proliferation of HaCaT cells but also exhibits potent healing activity in the mouse model of skin wounds [58,63]. As mentioned, WHP1 also contains this motif in its structure which seem to facilitate dimerization and formation of a glycine hinge, enhancing conformational freedom. This flexibility is essential for the switching of protein conformations between their active and inactive signaling states, such as receptor activation, kinase regulation, and potassium channel modulation [64].

WHP1 peptide demonstrated its wound healing potential by promoting the migration and proliferation of HaCaT keratinocytes, with the most significant response observed at a concentration of 62.5 µg/ml. It displayed remarkable potency in an *in vitro* wound scratch assay conducted on human skin keratinocytes in a dose-dependent manner, surpassing the wound healing activity of peptide LL-37, and also significantly induced proliferation of these keratinocytes. Notably, WHP1 exhibited no toxic effects on neither human red blood cells nor human keratinocytes even at the highest concentration tested. Not only keratinocytes, but numerous other types of cells and synchronized events, are implicated in wound healing. In these environments, growth factors and cytokines are primarily regulated by tissue macrophages which play a crucial role in multiple immune functions. A key issue in chronic wounds is the reduced levels of TGF-β, a growth factor essential for driving fibroblast-mediated fibronectin synthesis and collagen deposition. This is critical for effective extracellular matrix replacement during the remodeling phase of wounds [65,66]. Previous studies have shown that treatment with Ot-WHP markedly boosted TGF-β1 production in bone marrow-derived macrophages (BMDMs) and at wound sites, thereby accelerating wound healing in a mouse model with full-thickness wounds [10]. The WHP1 significantly elevates TGF-β1 secretion by macrophages, vital for the effective treatment of chronic wounds.

Chronic wounds have excessive and persistent levels of ROS. These can cause degradation of ECM proteins and impair the functioning of dermal fibroblasts and keratinocytes, and thereby hinder healing [67]. The WHP1 peptide also has antioxidant activity which efficiently neutralizes oxidants that exacerbate chronic wound conditions. This effect is primarily attributed to alkaline amino acids such as lysine in its sequence [68,69]. Numerous studies have explored the correlation between the *in vitro* activity and the *in vivo* efficacy and safety of wound healing peptides [10,59,68]. Our *in vitro* findings provide valuable insights into the wound healing efficacy and safety of WHP1. To further support its clinical development, we plan to assess its efficacy and safety in animal models. Additionally, we aim to investigate its combination with nanomaterials to enhance various aspects of the healing process and improve biocompatibility, inspired by the success of previous studies [70–72]. These efforts are directed toward establishing WHP1 as a promising therapeutic candidate for clinical application.

**Fig 7:**
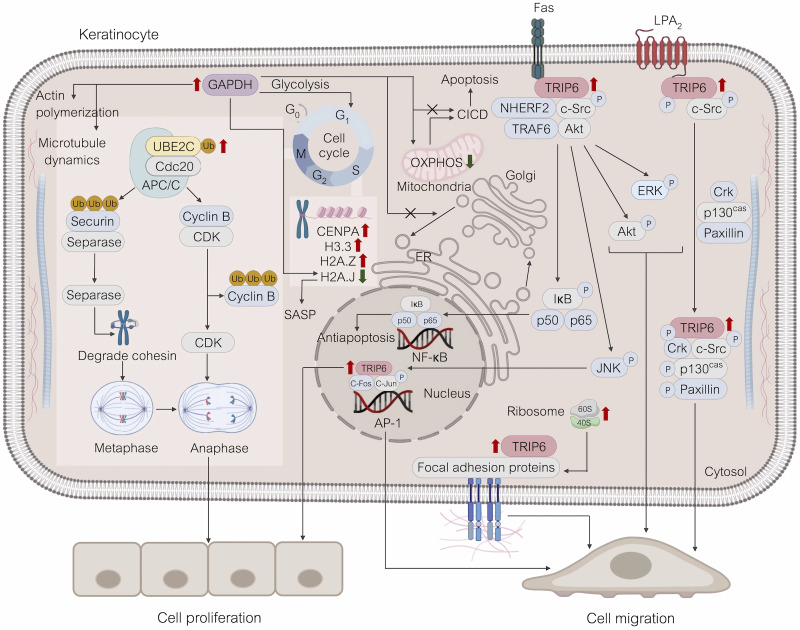
A diagram illustrating the alterations in protein expression and associated pathways in HaCaT cells following a 24-h treatment with WHP1. Red arrows indicate protein upregulation, while green arrows denote protein downregulation. This figure was created with BioRender (https://biorender.com/).

To comprehensively understand the intracellular mechanisms through which WHP1 peptide promotes cell proliferation and migration, proteomic profiling was applied for identifying alterations in protein expression in HaCaT keratinocytes following 24 h of peptide exposure (Fig 7). Our STRING analysis revealed that the upregulated proteins form a highly interconnected network. Notably, these proteins were significantly involved in cell cycle regulation and focal adhesion processes. Key upregulated proteins included the centromeric histone H3 variant CENPA and UBE2C, both crucial for cell cycle progression. UBE2C partners with the anaphase-promoting complex/cyclosome (APC/C) to catalyze the ubiquitination of critical cell cycle proteins, targeting them for proteasomal degradation. This degradation is important for the transition from metaphase to anaphase, and the exit from mitosis. Additionally, CENPA plays an important function in the proper epigenetic formation and function of the kinetochore, ensuring precise chromosome alignment and timely progression through mitosis. The coordinated activities of UBE2C and CENPA support cellular proliferation by ensuring proper chromosome segregation, maintaining genomic stability, preventing uncontrolled cell growth, and facilitating orderly cell cycle transitions [73,74].

To enhance cell migration, WHP1 also elevated the expression of focal adhesion proteins, notably TRIP6, alongside 40S ribosomes. TRIP6 plays crucial roles in cytoskeletal reorganization, regulating the assembly and disassembly of focal adhesions, and facilitating cell migration by acting as a scaffold for the recruitment of various proteins essential for these dynamic processes. Its interactions with a wide range of binding proteins support the dynamic changes required for cell movement [75]. In addition to TRIP6, 40S ribosomes preferentially accumulate at the leading edges of spreading cells, highlighting their significant involvement in the dynamics of focal adhesion [76]. These interconnected pathways indicate the importance of the upregulated proteins in promoting cellular proliferation, cell migration, and maintaining cellular architecture influenced by WHP1.

One of the upregulated proteins central to WHP1’s interaction network is GAPDH, a key glycolytic enzyme that is also involved in several non-glycolytic processes [77]. Upregulated GAPDH is a crucial coactivator for S-phase-dependent transcription, playing a key role in cell cycle progression [78,79]. GAPDH is recognized for its role in structural cellular processes, facilitating microtubule bundling and actin polymerization. These functions support cell movement and division. Moreover, catalytically active GAPDH may undergo transport within cells via microtubule treadmilling, potentially linking signal-stimulated glycolysis with cytoskeletal reorganization [77].

In proliferating cells, there is a substantial demand for energy, nutrients, and various biosynthetic activities to facilitate the transition from a non-dividing state and start duplication of cellular contents. This necessitates metabolic shifts from oxidative phosphorylation (OXPHOS) to glycolysis in response to nutrient levels. Notably, the rate of ATP production from glycolysis is significantly faster than OXPHOS, thus better supporting the high-energy requirements of rapidly dividing cells. Additionally, the extensive catabolism of glucose supplies ample glycolytic intermediates, such as sugar, glycerol, and nonessential amino acids, to fulfill the biosynthetic demands of rapidly dividing cells [80]. A downregulation of OXPHOS protein complexes involved in ATP generation was observed in HaCaT keratinocytes treated with WHP1. These include complex I, OGDHC, Cytc, LDH-A, ATP synthase subunit delta, TP-beta, MTX2, SAMM50, and TOMM40 [81]. Epidermal growth factor also orchestrates a wide array of metabolic activities, and these include glycolysis and glutamine metabolism. These processes play pivotal roles in coordinating essential cellular functions such as cell cycle regulation, apoptosis, cytoskeleton dynamics, and cell migration and invasion [82].

Apart from the previously discussed, GAPDH is implicated in modulating membrane trafficking and cellular signaling by regulating the dynamics of membrane fusion [83]. GAPDH-mediated transport inhibition allows cells to conserve energy by acting as a negative regulator of coat protein I (COPI) transport. This occurs through the binding of GAPDH to ADP-ribosylation factor 1 (ARF1), resulting in the inhibition of COPI and consequently halting transport from the Golgi. Therefore, GAPDH plays a crucial role in optimizing energy homeostasis and enhancing cell survival [84]. Clearly, the upregulation of GAPDH appears to facilitate the fulfillment of biosynthetic demands while conserving energy, as evidenced by the observed downregulation of cellular starvation within the STRING interaction network. This role highlights the remarkable adaptability of GAPDH in cellular energy management [85].

The phenomenon of replicative senescence in proliferating cells is marked by the progressive shortening of telomeres through successive cell divisions. This is driven by the production of ROS, which emerge as byproducts of cellular metabolic and redox reactions. Excessive oxidation can lead to proliferative arrest and oxidative damage. This process is triggered by stress and often results in an inflammatory phenotype. Senescent cells, collectively referred to as the Senescence-Associated Secretory Phenotype (SASP), secrete a diverse array of predominantly pro-inflammatory factors, potentially exacerbating tissue inflammation and degradation [86–88]. Certain reactive species can trigger apoptosis through the release of Cytc from mitochondria, which subsequently induces caspase activation [88]. Apart from Cytc, the histone variant H2A.J is present during replicative senescence and DNA damage and its accumulation may influence signaling pathways within senescent cells [86,87]. Interestingly, WHP1 apparently can lead to both damage and protection of cells. It was shown to enhance cell proliferation and migration through metabolic biosynthesis pathways which can lead to increased oxidative damage. But it also upregulated GAPDH, which provided safeguarding to caspase-independent cell death, and increased Bcl-xL expression, inhibiting pro-apoptotic proteins. In addition, WHP1 reduced the expression of the histone variant H2A.J, which is implicated in the SASP and oxidative stress-induced senescence. This reduction in H2A.J expression may lead to decreased oxidative stress, aiding in the elimination of free radicals. Further, it has the capability to scavenge oxidants on its own. These findings suggested that WHP1 has the ability to promote cell proliferation and migration while also reducing cellular senescence [60,84].

## Conclusion

Topical peptides offer a promising approach for enhancing wound healing outcomes. Through their targeting of specific biological processes involved in healing. The novel synthetic peptide, WHP1, exhibited considerable potential for enhancing the proliferation and migration of human skin keratinocytes by boosting the proteins involved in cell cycle regulation and focal adhesion. Complementary to this, it boosted glycolysis and mitigated oxidative stress thereby reducing cell senescence. Additionally, it increased secretion of TGF-β1 levels by macrophages. These findings indicate that WHP1 holds significant promise as a novel treatment for enhancing wound healing and pave the way for further exploration of peptides’ therapeutic potential in wound management.

## Supporting information

S1 File Table S1-S3. Details of the MIC of WHP1 peptide against 7 strains of human pathogenic Gram-positive and Gram-negative bacteria (Table S1), and the lists of upregulated (Table S2) and downregulated (Table S3) human proteins in WHP1-treated HaCaT keratinocytes for 24 h.
(DOCX)

## Abbreviations

BPs: bioactive peptides
HaCaT: human keratinocyte cell lines
CENPA: centromere protein A
UBE2C: ubiquitin-conjugating enzyme E2 C
TRIP6: thyroid receptor-interacting protein 6
GAPDH: glyceraldehyde-3-phosphate dehydrogenase
ROS: reactive oxygen species
TGF-β: transforming growth factor-β
