# Correction: Rational engineering unlocks the therapeutic potential of WHP1: A revolutionary peptide poised to advance wound healing

**DOI:** 10.1371/journal.pone.0335253

**Published:** 2025-10-22

**Authors:** Patcharin Khajornpipat, Onrapak Reamtong, Ratchaneewan Aunpad

There are errors in the Funding statement. The correct Funding statement is as follows: This research project is supported by National Research Council of Thailand (NRCT): (Contract No. N41A640212) awarded to PK, the Thailand Science Research and Innovation (TSRI) Fundamental Fund, fiscal year 2024 awarded to RA, and the Thammasat University Research Unit in Antimicrobial Agent and Application awarded to RA. There was no additional external funding received for this study.
