# Supplementary material for: Rational engineering unlocks the therapeutic potential of WHP1: A revolutionary peptide poised to advance wound healing
Source: PLoS One. 2025 May 14;20(5):e0323363. doi: 10.1371/journal.pone.0323363 (PMC12077786; doi:10.1371/journal.pone.0323363)
Supplement: S1 File — Table S1-S3. Details of the MIC of WHP1 peptide against 7 strains of human pathogenic Gram-positive and Gram-negative bacteria (Table S1), and the lists of upregulated (Table S2) and downregulated (Table S3) human proteins in WHP1-treated HaCaT keratinocytes for 24 h. (DOCX) [file pone.0323363.s001.docx]

**SUPPORTING INFORMATION**

**Rational Engineering Unlocks the Therapeutic Potential of WHP1: A Revolutionary Peptide Poised to Advance Wound Healing**

Patcharin Khajornpipat^1^, Onrapak Reamtong^2^, Ratchaneewan Aunpad^1,*^

^1^Graduate Program in Biomedical Sciences, Faculty of Allied Health Sciences, Thammasat University, Pathum Thani, Thailand; Email: aratchan@tu.ac.th

^2^Department of Molecular Tropical Medicine and Genetics, Faculty of Tropical Medicine, Mahidol University, Bangkok, Thailand; Email: onrapak.rea@mahidol.ac.th

*** To whom correspondence should be addressed:**

Assoc, Prof. Dr. Ratchaneewan Aunpad

Email: aratchan@tu.ac.th

Phone: +66829869213-9

**Determination of antimicrobial activity of peptide**

A modified version of the Clinical Laboratory and Standards Institute (CLSI) broth microdilution assay was employed to determine the minimum inhibitory concentrations (MICs) of the WHP1 peptide, following established methodologies previously published [1]. In this study, bacterial cultures were grown to mid-log phase and subsequently diluted to an initial inoculum concentration of 10⁷ CFU/ml in 20 ml of Mueller-Hinton broth (MHB) (Becton, Dickinson and Company, USA). The WHP1 peptide underwent two-fold serial dilution in PBS, spanning concentrations from 250 to 0.98 μg/ml. Equal volumes of the bacterial suspension and WHP1 peptide solutions were combined in a sterile 96-well plate and incubated at 37°C for 24 h with constant shaking at 220 rpm. The MIC values were determined as the lowest peptide concentration that completely inhibited visible bacterial growth [1]. LL-37, vancomycin, and gentamicin served as positive controls. All experiments were performed in triplicate.

**The WHP1 exhibits no antimicrobial activity**

The WHP1 peptide exhibited no antimicrobial activity against Gram-negative bacteria, including *Pseudomonas aeruginosa*, *Enterobacter cloacae*, *Acinetobacter baumannii*, and *Klebsiella pneumoniae*, nor against Gram-positive bacteria, such as *Staphylococcus aureus*, *Enterococcus faecium*, and *Staphylococcus epidermidis*, at the tested concentration range of 0.98–250 μg/ml.

Table S1. MIC of WHP1 peptide against 7 strains of human pathogenic Gram-positive and Gram-negative bacteria.

| **MIC (μg/ml)** | | | | |
| --- | --- | --- | --- | --- |
|  | **WHP1** | **LL-37** | **Vancomycin** | **Gentamicin** |
| Gram-negative bacteria | | | | |
| *Pseudomonas aeruginosa* DMST 15501 | >250 | 62.5 | >250 | 0.98 |
| *Enterobacter cloagae* DMST 553 | >250 | >250 | >250 | 0.98 |
| *Acinetobacter baumannii* ATCC 19606 | >250 | 50 | 125 | 31.25 |
| *Klebsiella pneumoniae* ATCC 27736 | >250 | 100 | >250 | 0.98 |
| Gram-positive bacteria | | | | |
| *Staphylococcus aureus* ATCC 25923 | >250 | >250 | 1.95 | 0.98 |
| *Enterococcus faecium* ATCC 700221 | >250 | 100 | >250 | >250 |
| *Staphylococcus epidermidis* ATCC 35984 | >250 | >250 | 3.91 | >250 |

**Table S2.** The lists of upregulated human proteins in WHP1-treated HaCaT keratinocytes for 24 h including those involved in the cell cycle and focal adhesion (The fold-change threshold of 2.0 with *p* ≤ 0.05).

| **No** | **Accession** | **Description** | **Fold change** | **GO—molecular function/biological process*** |
| --- | --- | --- | --- | --- |
| 1 | P04406 | Glyceraldehyde-3-phosphate dehydrogenase (G3P_HUMAN) | 2.08 | Playing crucial roles in both glycolysis and nuclear functions, contributing significantly to the organization and assembly of the cytoskeleton, as well as participating in pivotal nuclear processes, including transcription, RNA transport, DNA replication, and apoptosis |
| Cell cycle (HSA-1640170) | | | | |
| 2 | Q71DI3 | Histone H3.2 (H32_HUMAN) | 1.45 | Playing a crucial role in the regulation of transcription, DNA repair, DNA replication, and maintaining chromosomal stability |
| 3 | Q16695 | Histone H3.1t (H31T_HUMAN) | 1.45 | Playing a crucial role in the regulation of transcription, DNA repair, DNA replication, and maintaining chromosomal stability |
| 4 | P84243 | Histone H3.3 (H33_HUMAN) | 1.45 | Playing a crucial role in the regulation of transcription, DNA repair, DNA replication, and maintaining chromosomal stability |
| 5 | P68431 | Histone H3.1 (H31_HUMAN) | 1.45 | Playing a crucial role in the regulation of transcription, DNA repair, DNA replication, and maintaining chromosomal stability |
| 6 | Q9NPE3 | H/ACA ribonucleoprotein complex subunit 3 (NOP10_HUMAN) | 1.22 | Essential for both ribosome biogenesis and telomere maintenance |
| 7 | Q71UI9 | Histone H2A.V OS=Homo sapiens (H2AV_HUMAN) | 1.13 | A central role in transcription regulation, DNA repair, DNA replication, and chromosomal stability, while contributing to the formation of constitutive heterochromatin, and necessary for proper chromosome segregation during cell division |
| 8 | P0C0S5 | Histone H2A.Z OS=Homo sapiens (H2AZ_HUMAN) | 1.13 | A central role in transcription regulation, DNA repair, DNA replication, and chromosomal stability, while contributing to the formation of constitutive heterochromatin, and necessary for proper chromosome segregation during cell division. |
| 9 | O00762 | Ubiquitin-conjugating enzyme E2 C (UBE2C_HUMAN) | 1.09 | Controlling the progression of the cell cycle during mitosis, breaking down mitotic cyclins, regulating the activity of the anaphase-promoting complex, and facilitating cell proliferation |
| Focal adhesion (GO:0005925) | | | | |
| 10 | P46783 | Small ribosomal subunit protein eS10 (RS10_HUMAN) | 1.99 | An essential element of the 40S ribosomal subunit, responsible for coordinating the intricate process of protein synthesis within the cell |
| 11 | P61978 | Heterogeneous nuclear ribonucleoprotein K (HNRPK_HUMAN) | 1.52 | As major pre-mRNA-binding proteins and single-stranded DNA-bunding proteins, as well as playing a pivotal role in the p53/TP53 response to DNA damage, contributing to both transcriptional activation and repression |
| 12 | P61353 | Large ribosomal subunit protein eL27 (RL27_HUMAN) | 1.28 | A component of the large ribosomal subunit, which essential for the precise processing of rRNA, also known as 60S ribosomal protein L27 |
| 13 | P62277 | Small ribosomal subunit protein uS15 (RS13_HUMAN) | 1.15 | Alternatively known as 40S ribosomal protein S13, playing a crucial role in orchestrating protein synthesis within the cell |
| 14 | Q15654 | Thyroid receptor-interacting protein 6 (TRIP6_HUMAN) | 1.02 | A conduit for transmitting signals from the cell surface to the nucleus, facilitating the weakening of adherent junctions and promoting the reorganization of the actin cytoskeleton, thereby enhancing cell adhesion and migration |
| 15 | P62280 | Small ribosomal subunit protein uS17 (RS11_HUMAN) | 1.00 | Alternatively known as 40S ribosomal protein S11, playing a crucial role in orchestrating protein synthesis within the cell |

*Adapted from UniProt Knowledgebase

**Table S3.** The lists of downregulated human proteins in WHP1-treated HaCaT keratinocytes for 24 h including those involved in the Golgi-to-ER retrograde transport, senescence-associated secretory phenotype (SASP) and oxidative stress induced senescence, cellular response to starvation, as well as oxidoreductase and mitochondrial protein complex (The fold-change threshold of 2.0 with *p* ≤ 0.05).

| **No** | **Accession** | **Description** | **Fold change** | **GO—molecular function/biological process*** |
| --- | --- | --- | --- | --- |
| Golgi-to-ER retrograde transport (HSA-8856688) | | | | |
| 1 | P62820 | Ras-related protein Rab-1A (RAB1A_HUMAN) | -4.14 | Regulating vesicular protein transport from the endoplasmic reticulum (ER) to the Golgi apparatus and subsequently to the cell surface, as well as modulating the compact morphology of the Golgi |
| 2 | P84085 | ADP-ribylation factor 5 (ARF5_HUMAN) | -2.64 | A GTP-binding protein involved in protein trafficking, as well as modulating vesicle budding and uncoating within the Golgi apparatus |
| 3 | P61163 | Alpha-centractin (ACTZ_HUMAN) | -1.71 | Integral component of the ACTR1A/ACTB filament |
| 4 | Q9BUF5 | Tubulin beta-6 chain (TBB6_HUMAN) | -1.67 | Primary constituent of microtubules, composed of laterally associated linear protofilaments formed by α- and β-tubulin heterodimers |
| 5 | Q9H0U4 | Ras-related protein Rab-1B (RAB1B_HUMAN) | -1.61 | Key regulators of intracellular membrane trafficking, managing transport vesicle formation and membrane fusion, as well as essential for vesicular transport between the ER and Golgi compartments, crucial for maintaining Golgi morphology |
| 6 | P61204 | ADP-ribylation factor 3 (ARF3_HUMAN) | -1.53 | Involving in protein trafficking, modulating vesicle budding and uncoating within the Golgi apparatus |
| 7 | P84077 | ADP-ribylation factor 1 (ARF1_HUMAN) | -1.53 | Involving in protein trafficking, modulating vesicle budding and uncoating within the Golgi apparatus |
| Senescence-associated secretory phenotype (SASP) (HSA-2559582) and oxidative stress induced senescence (HSA-2559580) | | | | |
| 8 | P16104 | Histone H2AX (H2AX_HUMAN) | -1.50 | Essential for mediating the checkpoint-mediated arrest of cell cycle progression |
| 9 | P04908 | Histone H2A type 1-B/E (H2A1B_HUMAN) | -1.50 | Playing a central role in transcription regulation, DNA repair, DNA replication, and chromosomal stability |
| 10 | Q93077 | Histone H2A type 1-C (H2A1C_HUMAN) | -1.50 | Playing a central role in transcription regulation, DNA repair, DNA replication, and chromosomal stability |
| 11 | Q9BTM1 | Histone H2A.J (H2AJ_HUMAN) | -1.42 | Playing a central role in transcription regulation, DNA repair, DNA replication, and chromosomal stability |
| 12 | Q99878 | Histone H2A type 1-J (H2A1J_HUMAN) | -1.42 | Playing a central role in transcription regulation, DNA repair, DNA replication, and chromosomal stability |
| 13 | Q6FI13 | Histone H2A type 2-A (H2A2A_HUMAN) | -1.42 | Playing a central role in transcription regulation, DNA repair, DNA replication, and chromosomal stability |
| 14 | Q16777 | Histone H2A type 2-C (H2A2C_HUMAN) | -1.42 | Playing a central role in transcription regulation, DNA repair, DNA replication, and chromosomal stability |
| 15 | P20671 | Histone H2A type 1-D (H2A1D_HUMAN) | -1.42 | Playing a central role in transcription regulation, DNA repair, DNA replication, and chromosomal stability |
| Cellular response to starvation (HSA-9711097) | | | | |
| 16 | Q9UI12 | V-type proton ATPase subunit H (VATH_HUMAN) | -6.29 | Playing a role in acidifying and maintaining the pH of intracellular compartments, while also participating in clathrin-mediated endocytosis and contributing to the formation of endosomes |
| 17 | Q07020 | Large ribomal subunit protein eL18 (RL18_HUMAN) | -5.22 | A component of the large ribosomal subunit, which playing a crucial role in orchestrating protein synthesis within the cell, also known as 60S ribosomal protein L18 |
| 18 | P62249 | Small ribomal subunit protein uS9 (RS16_HUMAN) | -3.04 | Playing a crucial role in orchestrating protein synthesis within the cell, alternatively known as 40S ribosomal protein S60 |
| 19 | P05386 | Large ribomal subunit protein P1 (RLA1_HUMAN) | -1.95 | Playing a crucial role in the elongation step of protein synthesis, also known as 60S acidic ribosomal protein P1 |
| 20 | P62081 | Small ribomal subunit protein eS7 (RS7_HUMAN) | -1.61 | Playing a crucial role in orchestrating protein synthesis within the cell, alternatively known as 40S ribosomal protein S7 |
| 21 | P35268 | Large ribomal subunit protein eL22 (RL22_HUMAN) | -1.36 | A component of the large ribosomal subunit, which playing a crucial role in orchestrating protein synthesis within the cell, also known as 60S ribosomal protein L22 |
| 22 | P05387 | Large ribomal subunit protein P2 (RLA2_HUMAN) | -1.22 | Playing a crucial role in the elongation step of protein synthesis, also known as 60S acidic ribosomal protein P2 |
| 23 | P39023 | Large ribomal subunit protein uL3 (RL3_HUMAN) | -1.17 | A component of the large ribosomal subunit, which playing a crucial role in orchestrating protein synthesis within the cell, also known as 60S ribosomal protein L3 |
| 24 | Q6P5R6 | Ribomal protein eL22-like (RL22L_HUMAN) | -1.16 | A component of the large ribosomal subunit, also known as 60S ribosomal protein L22-like 1 |
| Oxidoreductase complex (GOCC:1990204) and mitochondrial protein complex (GOCC:0098798) | | | | |
| 25 | P49821 | NADH dehydrogenase [ubiquinone] flavoprotein 1, mitochondrial (NDUV1_HUMAN) | -6.05 | A core subunit of the mitochondrial membrane respiratory chain NADH dehydrogenase, which catalyzing the electron transfer from NADH through the respiratory chain using ubiquinone as an electron acceptor |
| 26 | O75431 | Metaxin-2 (MTX2_HUMAN) | -4.38 | Participating in the transport of proteins into the mitochondrion |
| 27 | P51970 | NADH dehydrogenase [ubiquinone] 1 alpha subcomplex subunit 8 (NDUA8_HUMAN) | -3.43 | An accessory subunit of the mitochondrial membrane respiratory chain NADH dehydrogenase or complex I |
| 28 | P55084 | Trifunctional enzyme subunit beta, mitochondrial (ECHB_HUMAN) | -2.79 | Essential for catalyzing the last three of the four reactions in the mitochondrial beta-oxidation pathway and breaking down fatty acids into acetyl-CoA through four consecutive reactions |
| 29 | P28331 | NADH-ubiquinone oxidoreductase 75 kDa subunit, mitochondrial (NDUS1_HUMAN) | -2.52 | Essential for catalyzing the entry and efficient transfer of electrons within the complex, serving as a core subunit of the mitochondrial membrane respiratory chain complex I, as well as playing a key role in the assembly and stability of complex I, contributing to the association of complex I with the ubiquinol-cytochrome reductase complex (complex III) to form supercomplexes |
| 30 | P99999 | Cytochrome c (CYC_HUMAN) | -1.47 | Functioning as an electron carrier, this protein transfers electrons to the cytochrome oxidase complex, which is the last protein carrier in the mitochondrial electron-transport chain, as well as interacting with Apaf-1 induces the activation of caspase-9, thereby promoting apoptosis |
| 31 | Q16795 | NADH dehydrogenase [ubiquinone] 1 alpha subcomplex subunit 9, mitochondrial (NDUA9_HUMAN) | -1.37 | An accessory subunit of the mitochondrial membrane respiratory chain NADH dehydrogenase or complex I |
| 32 | Q02218 | 2-oxoglutarate dehydrogenase complex component E1 (ODO1_HUMAN) | -1.26 | Playing a role as a component of the OGDHC, which contributing to the initial step of converting 2-oxoglutarate to succinyl-CoA and CO2, as well as holding significance in the Krebs (citric acid) cycle, a pivotal pathway for the oxidation of carbohydrates, fatty acids, and amino acids |
| 33 | O75874 | Isocitrate dehydrogenase [NADP] cytoplasmic (IDHC_HUMAN) | -1.23 | Critical for generating NADPH, a vital cofactor in numerous biosynthetic pathways |
| 34 | O43837 | Isocitrate dehydrogenase [NAD] subunit beta, mitochondrial (IDH3B_HUMAN) | -1.23 | Facilitating enzyme assembly and ensuring full activity in catalyzing the decarboxylation of isocitrate (ICT) into alpha-ketoglutarate |
| 35 | P00338 | L-lactate dehydrogenase A chain (LDHA_HUMAN) | -1.14 | Interconverting pyruvate and lactate simultaneously, while also facilitating the interconversion of NADH and NAD^+^ |
| 36 | P30049 | ATP synthase subunit delta, mitochondrial (ATPD_HUMAN) | -1.08 | Generating ATP from ADP in the presence of a proton gradient across the membrane |
| 37 | Q9Y512 | Sorting and assembly machinery component 50 homolog (SAM50_HUMAN) | -1.04 | Essential for maintaining the structure of mitochondrial cristae and ensuring the proper assembly of mitochondrial respiratory chain complexes |
| 38 | O96008 | Mitochondrial import receptor subunit TOM40 homolog (TOM40_HUMAN) | -1.00 | Channel-forming protein, which crucial for the import of protein precursors into mitochondria, assembly of the mitochondrial membrane respiratory chain complex I, and translocation of complex I components from the cytosol to the mitochondria |

*Adapted from UniProt Knowledgebase

**REFERENCE**

1. Steinberg DA, Hurst MA, Fujii CA, Kung AH, Ho JF, Cheng FC, et al. Protegrin-1: A broad-spectrum, rapidly microbicidal peptide with *in vivo* activity. Antimicrob Agents Chemother. 1997;41(8):1738-1742. doi:10.1128/AAC.41.8.1738. PMID: 9257752.
